# The Efficiency of the German Healthcare System: Some Puzzling Findings

**DOI:** 10.1002/hpm.70108

**Published:** 2026-07-29

**Authors:** Afschin Gandjour

**Affiliations:** ^1^ Frankfurt School of Finance & Management Frankfurt Germany

**Keywords:** cancer drugs, efficiency, Germany, healthcare system, waste

## Abstract

This article examines the cost‐effectiveness of the German healthcare system from the perspective of statutory health insurance (SHI). Using data for 2011–2014—the most recent period for which consistent national inputs are available—we compare a marginal, system‐wide cost‐effectiveness ratio with the cost‐effectiveness of newly approved cancer drugs. The analysis indicates that the German healthcare system incurred costs per life‐year gained that were broadly comparable to the incremental cost‐effectiveness ratios reported for many new cancer drugs. Sensitivity analyses varying the attribution of mortality reduction to healthcare do not materially alter this comparison. Although evidence of overuse and inefficiency in the German healthcare system is documented across multiple sectors, the available evidence does not clearly identify a single domain sufficiently large to explain the aggregate pattern. This raises the hypothesis that inefficiency may be diffuse, or that structural features common to high‐income health systems—such as diminishing marginal returns to health spending—play an important role. While the empirical estimates rely on data from roughly a decade ago, subsequent trends in treatment costs and life expectancy provide little indication that the underlying tension has dissipated. The findings highlight the need for more systematic cost‐effectiveness analysis across sectors, alongside rigorous evaluation of reforms and consideration of institutional and political constraints affecting implementation.

## Highlights

- System cost per life‐year ≈€90k—near new cancer drugs—suggests possible waste.
- Waste may be diffuse; pharma is unlikely the main driver.
- Adopt routine CEAs across sectors; consider mandatory CEAs for new drugs.
- Use rigorous evaluation of reforms to cut waste and inform global debates.

## 1 Introduction

Similar to other high‐income countries, the German healthcare system faces challenges in balancing health expenditures, outcomes, and accessibility to healthcare. In Europe, Germany's total spending on health as a percentage of the Gross Domestic Product (GDP) is relatively high (ranking first), while health outcomes in terms of life expectancy and other clinical endpoints are rather modest [1]. For example, life expectancy at birth and age 65 is only slightly above the European Union's average [1]. These outcomes are influenced by factors such as poor lifestyle choices, including smoking, excessive alcohol use, an unhealthy diet, and insufficient physical activity [1]. Given these factors, it would be questionable to directly infer that the cost‐effectiveness of the German healthcare system is poor [2].

Furthermore, the German healthcare system is characterised by simultaneous overuse (e.g., in terms of hospital care) [3] and underuse (e.g., in terms of new medicines). Potential explanations include incentives created by the diagnosis‐related group (DRG) payment system, fragmented healthcare delivery, defensive medicine, patient expectations and demand, and variation in adherence to evidence‐based clinical practice. Under the DRG system, hospitals are reimbursed on a per‐case basis, which creates incentives to increase admission volume rather than to provide additional services within a given admission. Thus, potential overuse may arise primarily at the case level (e.g., unnecessary hospitalizations or procedures) rather than through excessive service intensity within individual hospital stays.

This article aims to further investigate the cost‐effectiveness of the German healthcare system and to link it to existing overuse. While efficiency can be evaluated from a societal perspective encompassing total resource consumption, including costs borne by providers and patients, the present analysis explicitly adopts the perspective of statutory health insurance (SHI). It therefore focuses on payer expenditure as the relevant decision‐making context within the German healthcare system. Statements regarding the relevance or irrelevance of specific cost components are thus made with respect to SHI expenditure rather than overall societal resource use. This distinction is important, as conclusions about system‐level cost‐effectiveness may differ depending on the evaluative standpoint adopted. From this SHI perspective, newly published aggregated data on the system's cost‐effectiveness are presented and compared with the cost‐effectiveness of new cancer drugs in Germany. This comparison allows us to infer whether the German healthcare system is more or less cost‐effective than new cancer drugs, thus providing a new perspective on its overall value. New cancer drugs are used for the comparison because healthcare systems worldwide struggle with their high prices. In Germany, annual expenditures for new cancer drugs crossing the €100,000 mark have become increasingly common.

## 2 Cost‐Effectiveness of the German Healthcare System

A recent study from the perspective of the German social health insurance (SHI) [4] estimated a marginal, system‐wide cost‐effectiveness ratio for the German healthcare system. The analysis focused on 2011–2014, which was chosen because this is the most recent period for which the required published inputs were available in a consistent and comparable form. The cost‐effectiveness ratio was derived by relating intertemporal changes in lifetime healthcare spending to changes in life expectancy over this period. To estimate the contribution of healthcare to mortality reduction, the study used published data on mortality amenable to healthcare and all‐cause mortality, rather than applying a rule‐of‐thumb assumption about the share of life‐years gained attributable to medical care. Amenable (treatable) mortality was defined using the joint OECD–Eurostat lists of preventable and treatable causes, which are ICD‐10 based and apply an age threshold of under 75 years [5]. We use Eurostat's definition of ‘treatable’ (amenable) deaths: those that, given contemporary medical knowledge and technology, could largely have been avoided through timely and effective (i.e., optimal‐quality) health care.

For transparency, the system‐level estimate rests on four main steps. First, the model estimates age‐ and sex‐specific changes in remaining lifetime SHI spending between 2011 and 2014. Second, it estimates corresponding changes in remaining life expectancy. Third, it attributes the healthcare‐related share of mortality reduction using the ratio of changes in amenable mortality to changes in all‐cause mortality. Fourth, it divides the change in lifetime spending by the healthcare‐attributable change in life‐years. On this basis, the study found that approximately 48% of the mortality reduction in Germany during 2011–2014 was attributable to reductions in amenable mortality, implying a cost‐effectiveness ratio of roughly €90,000 per life‐year gained.

The interpretation of this estimate rests on several assumptions and design choices. It uses intertemporal changes in lifetime SHI spending and life expectancy as a pragmatic basis for estimating marginal cost‐effectiveness; relies on amenable mortality as an imperfect proxy for healthcare‐attributable survival gains; adopts the SHI perspective rather than a broader societal perspective; and reports life‐years rather than quality‐adjusted life‐years (QALYs) because consistent population‐level health‐related quality‐of‐life (HRQoL) weights are unavailable. The main sensitivity analysis varies the amenable‐mortality attribution parameter around the central estimate, while further interpretive checks consider HRQoL adjustment, alternative cancer‐drug survival denominators, and the persistence of post‐2014 trends. These calculations should therefore be interpreted as a transparent population‐level approximation rather than as a formal causal model with sampling‐based confidence intervals.

Changes in the age structure of the insured population are addressed by design, because the model uses age‐specific spending and survival and holds the population age distribution constant between periods. Similarly, changes in age‐specific utilization are not a source of bias per se: insofar as they contribute to reductions in amenable mortality, they are part of the marginal healthcare activity whose cost‐effectiveness the analysis seeks to capture. Secular increases in treatment intensity unrelated to survival gains are also not omitted from the analysis; they enter the spending numerator. If they do not generate survival or quality‐of‐life benefits, they appropriately worsen the estimated system‐level marginal cost‐effectiveness ratio (MCER). If they generate quality‐of‐life benefits, these may be missed in a life‐year‐based denominator, although available evidence suggests that HRQoL adjustment would be unlikely to overturn the central conclusion and could plausibly increase the estimated marginal cost‐effectiveness ratio [4].

As shown in the following section, it is useful to compare this SHI‐wide cost‐effectiveness ratio of roughly €90,000 per life‐year gained with the corresponding ratios for new cancer drugs.

## 3 Cost‐Effectiveness of New Cancer Drugs

In Germany, annual treatment costs for new cancer drugs launched between 2011 and 2015 that were granted an additional benefit by the Federal Joint Committee averaged €65,854 from the perspective of statutory health insurance (SHI) [6]. The corresponding average annual costs of comparators were €26,102, resulting in incremental costs of €39,751 [6].

Following Gandjour [7], evidence on the health benefits of these new cancer drugs is drawn from an analysis of anticancer drugs launched in Germany between 2011 and 2016 that had been granted an additional benefit by Germany's Federal Joint Committee by June 2016. The analysis reported a median incremental survival benefit of 4.7 months, or 0.39 years [8].

Following Gandjour [7], the cost‐effectiveness ratio of new cancer drugs is calculated by comparing incremental costs over 1 year with the reported survival gain. This yields an average estimate of €101,493 per life‐year gained (€39,751/0.39 life‐years). This figure should be interpreted as a central benchmark rather than as representative of all new cancer drugs. The median survival gain [8] summarises a heterogeneous set of drug–indication pairs, tumour types, mechanisms of action, and benefit categories, while the cost estimate reflects average annual incremental drug costs across different treatments. As a result, the implied incremental cost‐effectiveness ratio (ICER) obscures substantial distributional variation: some drug–indication pairs are likely to have ICERs well above this benchmark, whereas others may fall well below it. The comparison with the system‐level MCER should therefore be understood as a comparison of broad central tendencies rather than as implying that the cost‐effectiveness of individual cancer drugs clusters tightly around the reported estimate. This calculation is also limited to drug acquisition costs and therefore does not incorporate administration costs, the costs of managing adverse events, downstream savings from delayed tumour progression, or additional costs associated with prolonged survival. These cost components may partially offset one another—most notably downstream savings from delayed progression on the one hand, and administration, adverse‐event management, and prolonged‐survival costs on the other—but the net effect remains uncertain because it depends on regimen‐specific characteristics and may also reflect other factors not explicitly considered here.

A further concern is whether restricting costs to a 1‐year horizon understates total expenditures for cancers requiring prolonged therapy. Chen et al.‘s [9] analysis of drugs approved by the US Food and Drug Administration between 1995 and 2017 reports an average treatment duration of approximately 8.3 months, which is shorter than the 1‐year window applied here. Thus, in that sample, the 1‐year horizon would generally capture the average treatment course and is unlikely to substantially underestimate acquisition costs for the average drug.

Trial‐based median survival gains may, however, underestimate long‐term benefits because follow‐up is often limited. Using Chen et al.‘s extrapolated survival gain of 0.55 life‐years instead of the trial‐based median increases the denominator and reduces the ICER to €72,275 per life‐year gained (€39,751/0.55 life‐years). This extrapolated estimate should be interpreted as an external sensitivity benchmark rather than a directly German estimate. Its use assumes that extrapolated survival gains from the US FDA‐approved drug sample are broadly informative for the German AMNOG‐relevant cancer‐drug cohort, despite differences in regulatory context, tumour‐type mix, drug cohorts, and trial designs. Across these plausible survival estimates (0.39–0.55 life‐years), the implied ICER therefore ranges approximately from €72,000 to €101,000 per life‐year gained.

## 4 Wasteful Spending in the German Healthcare System

The German healthcare system incurs costs per life‐year gained that are remarkably similar in magnitude to those associated with new cancer drugs. Our central estimate of ≈€90,000 per life‐year gained is based on attributing 48% of the observed mortality reduction to healthcare‐amenable causes; varying that attribution to plausible alternatives, such as 40% or 60%, changes the estimate by roughly ± 20%, giving a range of ≈€75,000–€110,000 per life‐year gained. This range overlaps the cancer‐drug ICER range derived above, approximately €72,000–€101,000 per life‐year gained. However, this comparison should be interpreted cautiously. The system‐level MCER and cancer‐drug ICERs differ conceptually as well as empirically: the former is an aggregate, population‐based estimate derived from changes in SHI spending, life‐table survival, and amenable mortality, whereas the latter is an intervention‐level estimate based on incremental drug costs and trial‐based or extrapolated survival gains. The overlap between the ranges should therefore be interpreted as suggestive of broad comparability, not as evidence of formal equivalence. The two ranges are derived from different sources of uncertainty: the system‐level range reflects deterministic sensitivity analysis around the attributable share of mortality reduction, whereas the cancer‐drug range reflects alternative assumptions about survival gains, combining the trial‐based German estimate with an external US‐based extrapolated estimate. They therefore do not constitute statistically comparable confidence intervals. Because the system‐level estimate is derived from population‐level national accounts and life‐table data rather than from sample‐based regression models, it does not readily lend itself to conventional sampling‐based confidence intervals or formal equivalence testing.

Thus, even allowing for plausible alternative assumptions, the system‐level figure appears broadly comparable to the cost‐effectiveness ratios reported for many cancer drugs — a finding that is noteworthy given expectations of a substantial end‐of‐life premium [10].

Although standard health‐economic practice and international CEA norms favour reporting results in cost‐per‐QALY terms, our analysis reports cost per life‐year because consistent, population‐level HRQoL weights for the system‐wide estimate are not available; applying QALY weights to cancer drugs but not to the system would introduce methodological inconsistency. Empirical evidence indicates, however, that adjusting life‐years for health‐related quality of life typically does not substantially change ICERs for most cancer interventions [11]. By contrast, applying HRQoL adjustment to the German system‐level life‐years could plausibly increase the implied cost per QALY. The healthy life years (HLY) indicator is calculated using the Sullivan method, which combines life‐table mortality data with age‐specific prevalence of activity limitation derived from the EU‐SILC survey using the Global Activity Limitation Indicator. In this approach, the life‐table years lived at each age are weighted by the age‐specific proportion of individuals reporting no activity limitation. These weighted years are then summed over the remaining lifespan to estimate the number of healthy life‐years expected for an individual at birth, or at a given age. Consequently, if additional life‐years are increasingly lived with functional limitations, healthy life years increase more slowly than total life expectancy. Converting life‐years into QALYs using such population‐level quality adjustment would therefore likely reduce the system's QALY denominator more than the cancer‐drug denominator; accordingly, the finding that the system‐level cost per life‐year is comparable to many cancer‐drug ICERs would be unlikely to be overturned and may even be reinforced.

Whether this pattern has persisted beyond the 2011–2014 period cannot be established directly from the data used here. However, several subsequent developments suggest that the underlying tension may not have dissipated. The possibility that the broad comparison has persisted is supported by developments on both sides of the comparison. On the cancer‐drug side, annual treatment costs for new orphan and cancer drugs have continued to increase, while median survival improvement has plateaued at around 3–4 months. On the health‐system side, gains in life expectancy have declined—a trend that predates the onset of the COVID‐19 pandemic [12]—and healthy life expectancy stagnated between 2015 and 2022 based on Eurostat data [13], although some uncertainty remains due to breaks in the time series.

This interpretation assumes that the fraction of mortality classified as amenable to healthcare has remained broadly stable over time. While some change in this fraction cannot be excluded, the sensitivity analysis varying the attributable share by ± 20% suggests that moderate deviations from the central 48% attribution would not overturn the main comparison with new cancer‐drug ICERs. This range should not be interpreted as a formal confidence interval or as implying symmetric uncertainty. The true uncertainty may be distributional and directionally asymmetric, e.g. because of changes in cause‐of‐death coding, demographic ageing, shifts in disease composition, improvements in diagnostic attribution, or revisions to the OECD–Eurostat lists of treatable and preventable causes. Such factors could alter the measured amenable‐mortality share for reasons other than changes in the effectiveness of healthcare.

Although the simplified nature of the analysis needs to be acknowledged, results align well with the results of a similar analysis in the United States [14], thus confirming suspicions concerning substantial treatment overuse and waste in the German healthcare system.

A report by the OECD in 2017 identified three categories of waste: wasteful clinical care, operational waste, and governance‐related waste. Wasteful clinical care encompasses care leading to medical errors, ineffective and inappropriate treatments, and unnecessary duplication of services. Examples in the German healthcare system are thyroid operations, ultrasound examination of the ovaries, and prescription of proton‐pump inhibitor drugs [15]. Operational waste occurs when excessive prices are paid or unused inputs are discarded. Governance‐related waste pertains to ineffective administrative expenditure. Current estimates suggest that waste and overuse in international healthcare systems account for 20% to a third of total healthcare spending [16]. Notably, estimates for the United States are not substantially higher [17], despite its higher health expenditure as a percentage of GDP compared to other developed countries.

Applying these waste estimates to Germany indicates that a more efficient healthcare system could produce one additional life year at a cost of €63,777 to €76,533. This figure remains surprisingly high and exceeds the known ICERs of many treatments in Germany, such as pharmaceuticals [18]. Given Germany's high spending on healthcare as a percentage of GDP, it is plausible that the extent of waste in the German healthcare system might be higher than indicated by these international estimates. Consequently, a thorough investigation of different healthcare sectors is necessary to identify areas of extensive waste (i.e., above international estimates) in terms of the three categories of waste.

Regarding the pharmaceutical sector, it is important to note that, compared with new oncological drugs, many medicines—especially generics—are associated with substantially lower treatment costs. In 2019, the average prescription cost for a generic drug was €33.92, whereas the average net cost of a patent‐protected drug prescription was €471.50 [19]. Despite this, inefficiencies persist in the pharma sector due to the overuse of medicines (i.e., unnecessary care), underutilisation of generic and biosimilar alternatives, and the issue of discarded pharmaceuticals. Overuse is more likely to occur with generics than with patent‐protected drugs because of their low prices. However, the budgetary relevance of such overuse is likely to be limited, as generics account for only approximately 7% of total SHI pharmaceutical expenditure [20]. By contrast, inefficiencies related to patent‐protected medicines are likely to arise less from overuse than from limited uptake of lower‐cost alternatives and, in some cases, from high prices. Consistent with this, uptake among eligible patients in subgroups with confirmed added benefit averages below 20%, implying substantial underuse relative to full uptake in the indicated population; at the same time, observed use may already include some degree of overuse [21]. Furthermore, the potential savings from generic and biosimilar substitution are estimated to be only around 1% of total SHI expenditure [22, 23]. Spending on discarded drugs is even smaller. Thus, although inefficiencies exist in the pharmaceutical sector and high‐priced patented medicines may contribute to the unfavourable cost‐effectiveness ratio of the German healthcare system, their limited uptake suggests that this sector is unlikely to be the primary source of waste in the healthcare system. Additional areas requiring scrutiny include the medical equipment sector, the hospital sector, the outpatient sector, administrative expenses, and workforce inefficiency.

There is evidence suggesting an overuse of medical equipment in Germany. The country conducts the highest number of in‐hospital MRI scans per 1000 population among OECD countries [24]. Additionally, a notable variation exists in the use of medical imaging procedures in hospitals across different German regions, which is not entirely accounted for by the regional burden of disease, especially in the case of CT procedures [25]. By contrast, the number of in‐hospital CT scans per 1000 population in Germany is roughly in line with the OECD average [24].

With regard to the hospital sector, it constitutes the largest component of healthcare expenditure in Germany. Current estimates of overuse in hospitals are broadly consistent with those reported for international healthcare systems [26]. Therefore, even after adjusting for waste, these estimates do not fully explain the high cost‐effectiveness ratio of the German healthcare system. Although inpatient case numbers have declined since 2016 [27], this trend does not necessarily indicate a structural reduction in waste. The decline was partly driven by the COVID‐19 pandemic and therefore does not provide clear evidence that low‐value care has been systematically eliminated. In any case, it is important to acknowledge that estimates of overuse in German hospitals are subject to considerable uncertainty.

From a societal perspective, operational inefficiencies in hospitals affect real resource use and thus overall system efficiency. However, the present analysis adopts the perspective of the SHI and focuses on payer expenditure rather than total resource consumption. Under Germany's DRG‐based reimbursement system, operational inefficiencies are largely borne by hospitals rather than directly by SHI, as reimbursement is primarily case‐based rather than cost‐based.

Medical errors in hospitals may be another source of wasteful clinical care. There is a huge gap between the officially acknowledged number of medical errors (2377 in 2022) [28] and the unreported rate, estimated to affect one percent of hospital cases [28]. Nevertheless, medical errors may not directly affect SHI's expenditure, given that some DRGs incorporate penalties for hospitals with higher‐than‐expected readmission rates, which can be linked to medical errors. However, medical errors can require additional treatment or long‐term care that is not fully covered by the original DRG payment. This may generate additional costs for payers, particularly when follow‐up care is provided in other settings, such as skilled nursing facilities or outpatient care.

The extent of wasteful clinical care in the German outpatient sector remains a subject of debate, with some arguing it is more ambiguous than in other sectors. Common critiques point to practices such as ordering excessive tests (e.g., imaging tests), overprescribing medications, unnecessary surgeries, and delivering care that lacks a solid evidence base, such as the so‐called IGeL‐Leistungen (which are not covered by the SHI, however). Nevertheless, for procedures that can be safely delivered in both the outpatient and inpatient settings—such as selected surgical interventions—outpatient care may be more cost‐effective because of lower fixed costs and the absence of expenses associated with overnight stays.

Administrative costs in the healthcare system incur at different levels, including payers, institutions, and individuals. These costs are therefore not restricted solely to the SHI. SHI‐level administrative costs contribute only 4% of SHI expenditure [30]. However, administrative costs at the level of institutions and individuals are not relevant from an SHI perspective and, therefore, cannot be responsible for explaining the observed similarity between the cost‐effectiveness of single interventions and the overall healthcare system from an SHI standpoint.

Workforce inefficiency may represent a significant problem in the German healthcare system and may overlap with both administrative inefficiency and hospital inefficiency. For example, inefficient administrative processes may make it more difficult for hospitals to recruit and retain staff, contributing to understaffing and lower productivity. Inefficient care‐delivery processes may also increase staff workload and the risk of errors. Estimating the exact cost of workforce inefficiency is challenging, and available global benchmarks are dated and uncertain. The World Health Report [31] suggested an inefficiency rate of 15%–25%, corresponding to worldwide costs exceeding US$500 billion annually. While this estimate indicates that workforce inefficiency may be quantitatively important, its vintage and global scope limit its direct applicability to contemporary Germany. For Germany, the available evidence therefore supports only the more cautious conclusion that workforce inefficiency may be budgetarily relevant, but its magnitude cannot be estimated reliably from these global benchmarks alone.

Taken together, these sectoral observations suggest that the high system‐level cost per life‐year gained is unlikely to be explained by a single dominant source of waste. Rather, the evidence points to a combination of mechanisms operating across sectors. In pharmaceuticals, the coexistence of high‐priced patented medicines, underuse of some innovations, and limited budgetary gains from generic or biosimilar substitution illustrates how high‐value and low‐value care can coexist within the same sector. In hospitals and outpatient care, overuse, variation in practice patterns, and uncertainty around the magnitude of waste point to diffuse marginal inefficiencies rather than one clearly isolable source. Administrative and workforce inefficiencies further suggest that part of the problem may arise from system‐level frictions and fragmented governance rather than from clinical overuse alone. These patterns help explain why the aggregate MCER can appear unfavourable even when no individual sector obviously accounts for the full discrepancy.

## 5 Summary and Conclusions

Data from nearly a decade ago revealed a concerning pattern: during the period studied, the German healthcare system incurred costs per life‐year gained that were nearly on par with those associated with new cancer drugs. This issue, rooted in healthcare waste, has likely persisted over time rather than representing a transient trend. Equally important, however, is the difficulty of identifying a single sector or mechanism that could, on current evidence, fully account for the relatively high system‐wide cost per life‐year gained. Although overuse and inefficiency are documented across multiple domains—pharmaceuticals, hospitals, imaging, workforce, and administration—the magnitude of estimated waste within each individual sector does not obviously approach the 20%–33% range often cited in international comparisons.

The difficulty in locating large concentrations of waste may stem from several factors. First, inefficiency may be embedded in countless marginal decisions—such as slightly excessive diagnostic testing, minor deviations from clinical guidelines, or small administrative frictions—that are individually modest but collectively substantial. Second, governance in the German healthcare system is fragmented, with costs and benefits distributed across multiple actors. Third, high‐value and low‐value interventions frequently coexist within the same sector, so aggregate sectoral indicators may obscure important internal heterogeneity. Finally, some structural features—such as high input prices or diminishing marginal returns to medical innovation—may raise system‐level cost‐effectiveness ratios without appearing as clearly identifiable waste within sector‐specific accounting.

This tension is itself informative. It suggests that inefficiency may be diffuse and institutionally dispersed rather than concentrated in a single dominant area amenable to straightforward correction. Alternatively, the elevated system‐wide ratio may partly reflect structural features common to high‐income health systems rather than uniquely German institutional shortcomings. Notably, a similar comparison for the United States revealed a broadly comparable pattern despite markedly different financing and delivery structures. This parallel suggests that part of the phenomenon may be inherent to modern high‐technology medicine, particularly diminishing marginal returns to health spending and the rising cost of marginal survival gains.

Additional context is provided by the growing empirical literature estimating country‐level cost‐effectiveness thresholds using econometric, supply‐side methods. These thresholds reflect the marginal productivity of healthcare spending—or, equivalently, the health opportunity costs associated with reallocating healthcare resources. Studies for the United Kingdom, Spain, and the Netherlands [32, 33, 34] suggest that empirically derived health‐opportunity costs can differ substantially across countries and often lie below traditional rule‐of‐thumb benchmarks. At the same time, Germany's recent model‐based threshold estimate of approximately €90,000 per life‐year gained is higher than several empirical estimates reported for other European systems. If interpreted as a supply‐side threshold for Germany, this figure would imply that new life‐prolonging technologies with ICERs below this level could be adopted without reducing overall health‐system efficiency, whereas technologies above this level would require either price reductions, stronger evidence of additional benefit, or justification on other grounds, such as severity, rarity, or end‐of‐life considerations. In the context of drug reimbursement, formal adoption of such a threshold would therefore strengthen the link between AMNOG benefit assessment, price negotiation, and opportunity‐cost‐based resource allocation. However, it could also create upward pricing incentives if manufacturers view the threshold as a target rather than a ceiling, and it would raise difficult institutional questions about whether Germany is willing to make reimbursement decisions explicitly contingent on cost‐effectiveness. Direct comparisons with other countries are further complicated by methodological differences, including differences in outcome measurement—for example, the use of mortality‐based measures rather than QALYs, which may omit important health benefits such as quality‐of‐life gains—as well as alternative identification strategies and differences in data periods. It therefore remains unclear whether observed differences reflect genuine cross‐country variation in marginal productivity and inefficiency, or primarily differences in methods and assumptions. Harmonised cross‐country analyses using consistent definitions of amenable mortality, comparable spending data, similar outcome measures, and overlapping time periods would be required to determine whether the observed pattern reflects Germany‐specific institutional inefficiency, differences across countries in the health gains produced by additional healthcare spending, or structural features common to high‐income, technology‐intensive health systems.

Despite its long‐standing nature, the issue of waste has received relatively limited attention in Germany. For many years, the German healthcare system was slow to acknowledge the widespread occurrence of waste and its significant impact on healthcare expenditure [35]. This lack of attention may have allowed the problem to persist and possibly intensify over time, thereby increasing the need for reform.

Recognising the imperative to address this issue, the German Ministry of Health has initiated several measures to reduce waste in the healthcare system. However, the delayed recognition of the problem has inevitably hampered the ability to effectively address it. Early recognition and intervention could have led to more timely and efficient mitigation strategies.

The delayed acknowledgement of the waste issue highlights the importance of proactive monitoring and analysis of healthcare systems. Regularly examining data and identifying trends can help pinpoint areas of inefficiency and waste, enabling timely intervention and improvement.

To effectively address this persistent issue of healthcare waste in the German healthcare system, it is imperative to adopt a more systematic and forward‐thinking approach—an aim that aligns with a key recommendation from the World Health Report [31]. To shed light on areas with potential inefficiencies and develop targeted solutions, future research should prioritize the implementation of rigorous cost‐effectiveness analyses (CEAs) across various sectors and disease areas. By conducting CEAs, future research endeavors can embark on a journey to uncover the inner workings of the healthcare system's 'black box' of waste. A related step involves the introduction of mandatory CEAs for new innovative drugs, a practice currently not obligatory in Germany, unlike many other European countries. This proactive approach aims to deepen our understanding of the observed discrepancy between the cost‐effectiveness of individual interventions and the overall healthcare system, as revealed in this analysis.

However, the implementation of routine, system‐wide cost‐effectiveness analysis in Germany faces significant political economy constraints. The German healthcare system is characterized by corporatist governance structures, in which statutory health insurers, provider associations, hospitals, and pharmaceutical manufacturers share regulatory authority. These stakeholders often have divergent interests and substantial veto power, making centralized cost‐effectiveness–based rationing politically sensitive. While Germany conducts early benefit assessments for new drugs under AMNOG, reimbursement and pricing are not formally tied to explicit cost‐effectiveness thresholds, and mandatory economic evaluations—common in several other European countries—have historically faced resistance. Concerns about implicit rationing, distributive conflicts, and potential loss of stakeholder autonomy have limited the political feasibility of binding CEA rules. Advancing routine CEA would therefore likely require not only further methodological refinement in the estimation of cost‐effectiveness thresholds, but also broader institutional support, including clearer principles for resource allocation and better alignment of incentives across corporatist actors.

More generally, while CEA and Health Technology Assessment (HTA) can support more transparent and evidence‐informed resource allocation, the broader literature does not clearly establish that HTA implementation necessarily generates savings at the healthcare‐system level [36]. This evidence is not Germany‐specific and therefore should be interpreted only as a general caution against assuming that more systematic HTA would automatically reduce aggregate expenditure. Even when HTA finds an intervention to be cost‐effective, aggregate spending can still rise if approval expands the treated population, encourages earlier or broader use, or if higher treatment volumes offset reductions in per‐patient prices.

In addition to the recommended implementation of CEAs, several innovative policy ideas have been proposed to contribute to waste reduction and efficiency improvement in the German healthcare system. These ideas encompass leveraging technology and data analytics, enhancing patient involvement, optimizing payment models, and fostering collaboration across the healthcare landscape. Inevitably, any such policy measure needs to be thoroughly evaluated—an effort that extends beyond the scope of traditional HTA. We recommend prioritizing experimental and quasi‐experimental designs (cluster randomized or stepped‐wedge rollouts, difference‐in‐differences, interrupted time series, synthetic controls, and regression discontinuity where applicable) and embedding economic evaluation (cost‐effectiveness and budget‐impact analyses) alongside process and implementation measures. Natural experiments created by differential implementation across Länder—for example, pilot bundled‐payment schemes, regional integrated‐care contracts, or hospital‐planning reforms—offer especially attractive opportunities for causal inference.

High‐quality empirical evidence is essential not only for national decision‐making but also for enabling countries to learn from each other's policy experiments. Clear insights into what works—and what does not—can support evidence‐informed policymaking and foster international learning. By implementing these innovative policies alongside existing measures—and ensuring their systematic evaluation—Germany can take proactive steps toward reducing healthcare waste and enhancing the overall efficiency and cost‐effectiveness of its healthcare system. This multifaceted approach may contribute to better resource allocation and improved patient outcomes.

## Funding

The author has nothing to report.

## Ethics Statement

The author has nothing to report.

## Conflicts of Interest

The author declares no conflicts of interest.

## Data Availability

Data sharing not applicable to this article as no datasets were generated or analysed during the current study.
